# Editorial: Care of the extremely preterm infant

**DOI:** 10.3389/fped.2025.1751915

**Published:** 2026-01-13

**Authors:** Qiuping Li, Rong Ju, Fu-Sheng Chou

**Affiliations:** 1 Department of Pediatrics, Seventh Medical Center of The Chinese PLA General Hospital, Beijing, China; 2 NICU, Bayi Children's Hospital, Beijing, China; 3 NICU, Chengdu Women and Children's Central Hospital, Chengdu, China; 4 Southern California Permanente Medical Group, Yorba Linda, CA, United States; 5 Kaiser Permanente Bernard J. Tyson School of Medicine, Pasadena, CA, United States

**Keywords:** nursing care, preterm infant, treatment, prognosis, progress

Extremely preterm infants (EPIs), who are born before 28 weeks of gestation, represent one of the most vulnerable populations in neonatal medicine. They are at a relatively high risk of mortality, acute complications, and long-term morbidities spanning physical, cognitive, and social domains. Despite remarkable advances in neonatal care over the past decades, optimizing their outcomes remains a formidable clinical challenge that requires integrated insights from clinical research, innovative care delivery, and family-centered practices. The Research Topic “*Care of the Extremely Preterm Infant*” was conceived to address this gap by curating cutting-edge studies that illuminate key aspects of prevention, intervention, prognosis, and long-term management. After a rigorous peer-review process, 15 articles were selected for publication, collectively offering a comprehensive perspective on the multifaceted care of EPIs across diverse clinical and socioeconomic contexts.

## Therapeutic interventions: targeting acute complications

A core focus of the Research Topic lies in identifying effective interventions to mitigate acute neonatal complications, which are critical determinants of short-term survival and long-term health. Yao et al.'s original research provided compelling evidence that early low-dose hydrocortisone administration not only reduces the risk of Grade II+ bronchopulmonary dysplasia (BPD) but also shortens the duration of non-invasive ventilation in infants born before 26 weeks of gestation. This finding is particularly significant because it offers an actionable strategy to improve respiratory outcomes, knowing that BPD is one of the leading causes of chronic respiratory morbidity in EPIs. Park et al. developed a C5.0 decision tree model to predict massive pulmonary hemorrhage (MPH) and MPH-associated mortality in extremely low birth weight infants (ELBWIs) with gestational age ≤25 + 2 weeks and a 5-min Apgar score ≤7, with these factors emerging as key discriminators. The model's high predictive accuracy (AUC >88%) enables the early identification of high-risk infants, thus facilitating timely interventions to reduce mortality.

A retrospective study by Suh et al. addressed another critical issue in neonatal nutrition: hypertriglyceridemia (HTG) in preterm infants under 32 weeks of gestation. The authors identified lower birth weight, treatment for patent ductus arteriosus, and higher intake of intravenous lipid emulsions as significant risk factors for HTG, demonstrating that reducing lipid doses accelerates triglyceride normalization and lowers the risk of severe retinopathy of prematurity. Meanwhile, a prospective study by Alanazi et al. clarified the correlation between the Oxygenation Index (OI) and the Oxygen Saturation Index (OSI) in EPIs, showing that measurements within the first 24 h predict early mortality, and that daily mean values from days 4–7 forecast BPD at 36 weeks postmenstrual age. Together, these studies advance precision medicine in neonatal care by equipping clinicians with tools to tailor interventions and monitor responses.

## Prognostic assessment: contextualizing survival and morbidity

Survival and morbidity outcomes for EPIs vary widely across healthcare settings, highlighting the need for context-specific data to guide care policies. Rodriguez-Sibaja et al.'s original research filled a critical gap by evaluating EPIs born between 22.0 and 27.6 weeks in a middle-income setting by using multiple denominators to provide a comprehensive survival assessment. Their findings underscore the challenges of resource-limited environments and offer valuable benchmarks for regions with similar socioeconomic profiles. Similarly, Kalyanam et al. investigated 1,627 ELBWIs across three U.S. NICUs and found that the center-specific variations in survival and severe intraventricular hemorrhage are attributable to differences in care practices, such as resuscitation decisions. This emphasizes the importance of evidence-based practice sharing to standardize and improve care quality.

A network analysis conducted by Peterson et al. on periviable infants born between 22^+0^ and 23^+6^ weeks of gestation offered novel insights into mortality timing. The study reveals that 65% of infants born at 22 weeks and 52% of those born at 23 weeks who died did so within the first seven days of life. Furthermore, 89% of the 22-week-old infants who died succumbed by day 14. This supports a “trial of therapy” approach that balances the potential for survival against the risk of iatrogenic harm. Collectively, these prognostic studies highlight the need to contextualize outcomes within the framework of healthcare resources, care practices, and gestational age, informing both clinical decision-making and health policy.

## Long-term health and developmental trajectories

The impact of extreme preterm birth extends far beyond the neonatal period, necessitating long-term monitoring and proactive management. A narrative review by Gette et al. comprehensively delineated the long-term complications of preterm birth, spanning the neurodevelopmental, respiratory, cardiovascular, renal, gastrointestinal, and endocrine systems. By synthesizing the current literature, the review equips healthcare providers with a holistic understanding of lifelong risks, emphasizing the value of long-term follow-up programs. To complement this, Wang et al. conducted a real-world observational study of 79,514 Chinese preterm infants and established growth curves for 0–36 months of corrected age, revealing that very preterm infants consistently lagged behind term standards in weight and length, even at 36 months. Their study addresses the lack of specialized growth benchmarks for preterm infants, providing a critical tool for assessing postnatal development.

## Care delivery: enhancing family-centered and trauma-informed practices

Optimal care for EPIs requires a multidisciplinary approach that integrates clinical expertise with family needs and psychological support. Peterson et al.'s rapid review and thematic analysis of periviable counseling practices identified three core principles - Transparency, Collaboration, and Empowerment - that align with trauma-informed care. Their findings highlight the importance of honest and individualized communication with parents, including discussions about comfort care, to build trust and support informed decision-making. A companion study by Peterson et al. compared international guidelines for periviable infants across 10 high healthcare-spending countries. The authors noted variations in viability definitions (22 + 0–25 + 6 weeks) and delivery room management, while emphasizing the need for structured shared decision-making tools. A further study by Peterson et al. explored parent and perinatal professional priorities in pre-birth conversations about periviable infants through a thematic analysis of semi-structured interviews, which further enriches our understanding of effective communication strategies.

A clinical observational study by Zhan et al. identified the 1-min Apgar score and gestational age as contextual factors influencing pain responses, adding a critical dimension to neonatal care. Infants with lower Apgar scores and younger gestational age often exhibit less pronounced pain responses, which challenges the reliability of observation-based pain assessment alone. This study advocates integrating physiological markers into pain evaluation to improve care quality. Together, these articles advance family-centered and trauma-informed care, highlighting the pivotal roles of communication, psychological support, and holistic assessment in optimizing outcomes.

## Specialized case management and clinical challenges

Two case reports in the Research Topic addressed rare but clinically significant complications, offering practical insights for frontline clinicians. Hooper et al. described a delayed recurrence of staphylococcal scalded skin syndrome (SSSS) in an ELBWI, which was initially misdiagnosed as a yeast infection, emphasizing the need for multidisciplinary teams to manage complex infections in immunocompromised preterm infants. A case report by Calandrino et al. reported an elevated methemoglobin level in a preterm infant treated with glyceryl trinitrate (GTN) patches for limb ischemia, underscoring the importance of monitoring and exercising caution while administering GTN therapy, especially to infants with immature skin and enzyme systems. These cases provide valuable lessons for the diagnosis and management of rare complications, filling gaps in clinical knowledge.

## Conclusion

The 15 articles cited in this Research Topic broaden our understanding of the care of EPIs by addressing therapeutic innovation, prognostic contextualization, long-term health, family-centered practices, and specialized case management. By integrating insights from diverse settings—from middle-income countries to high-resource NICUs—and across the care continuum, the Research Topic offers a roadmap for improving outcomes. Key priorities emerging from these studies include precision interventions tailored to individual risk profiles, evidence-based practice standardization, long-term follow-up programs, trauma-informed family counseling, and vigilance for rare complications. As neonatal care continues to evolve, this Research Topic underscores the importance of collaborative, multidisciplinary research to address the unique challenges of EPIs, ultimately striving for equitable and high-quality care that optimizes both survival and quality of life.

